# Restructured GEO: restructuring Gene Expression Omnibus metadata for genome dynamics analysis

**DOI:** 10.1093/database/bay145

**Published:** 2019-01-16

**Authors:** Guocai Chen, Juan Camilo Ramírez, Nan Deng, Xing Qiu, Canglin Wu, W Jim Zheng, Hulin Wu

**Affiliations:** 1 School of Biomedical Informatics, University of Texas Health Science Center at Houston (UTHealth), Houston, Texas, USA; 2 Department of Biostatistics & Data Science, School of Public Health, University of Texas Health Science Center at Houston (UTHealth), Houston, Texas, USA; 3 Department of Biostatistics and Computational Biology, School of Medicine and Dentistry, University of Rochester, Rochester, New York, USA; 4 TechWave International. Inc., Houston, Texas, USA; 5 Universidad Antonio Nariño, Bogotá, Colombia

## Abstract

**Motivation:**

Gene Expression Omnibus (GEO) and other publicly available data store their metadata in the format of unstructured English text, which is very difficult for automated reuse.

**Results:**

We employed text mining techniques to analyze the metadata of GEO and developed Restructured GEO database (ReGEO). ReGEO reorganizes and categorizes GEO series and makes them searchable by two new attributes extracted automatically from each series’ metadata. These attributes are the number of time points tested in the experiment and the disease being investigated. ReGEO also makes series searchable by other attributes available in GEO, such as platform organism, experiment type, associated PubMed ID as well as general keywords in the study’s description. Our approach greatly expands the usability of GEO data, demonstrating a credible approach to improve the utility of vast amount of publicly available data in the era of Big Data research.

## Introduction

In this Big Data era, data-driven approach for biomedical research becomes more and more important (1), and data sharing and reuse has received a great deal of attention in the scientific community (2, 3). However, many barriers still exist for reuse of others’ data (4). In particular, when a public data repository or database is not designed for data reuse, the metadata (data about data) may be vague and documented in plain text, and not structured and well standardized, making it difficult for researchers to identify the data they need. Using text mining techniques to identify and organize critical metadata information from the data repositories or databases can greatly increase the reuse of these shared data.

The Gene Expression Omnibus (GEO) is the largest data repository designed for archiving and distributing microarray, next-generation sequencing, and other functional high-throughput genomics data (1, 5, 6). As of January 2018, the GEO repository hosts over 90 000 series submitted directly by an estimated 26 000 laboratories, comprising over 2 × 10^6^ samples (https://www.ncbi.nlm.nih.gov/geo/summary) derived from over 3000 organisms. GEO offers a simple submission procedure that allows researchers to summarize a study in plain text. However, such flexibility has also resulted in unstructured metadata in plain English scattered in different sections of each study’s description making its reuse difficult.

**Figure 1:**
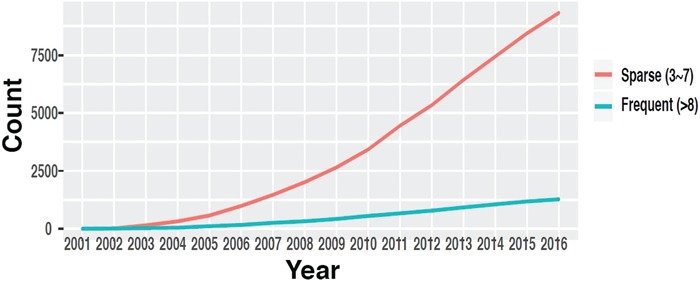
The number of datasets in GEO with multiple time points. (source: GEOMetadb).

Time-course data are fundamental to study genome dynamics and identify genes whose expression changes significantly over a defined period of time. Discovery of these genes is crucial in order to understand underlying disease mechanisms, detect novel targets for intervention and improve prevention and treatment of diseases (7–9). While robust statistical methods have been developed to identify these genes and discover dynamic gene regulatory networks (10–13), these methods generally require time-course data with a certain minimum number of time points to yield reliable results. Even though the availability of time-course data in GEO has increased throughout the years (14) (Figure 1), such information is mostly buried in the metadata in the form of unstructured plain text and is very difficult to obtain, especially through automated means.

To maximize reuse, GEO incorporates a comprehensive search function to discover experimental results through query terms such as `organism’ or `cell type’ (15). Additional tools such as RESTful APIs (16, 17) and GEOmetadb (14) can also assist in retrieving data from GEO. However, important information such as the time course of a study still cannot be obtained, making it extremely difficult to reuse GEO data to study genome dynamics. Many different approaches attempted to enhance the retrieval of data from GEO database. Aside from GEOmetadb (14), other previous efforts include GEOquery (18), which offers an interface between BioConductor (19) and GEO for the retrieval of records through the same fields available in this repository. More recent works include ScanGEO (20) and ImaGEO (21), which offer web interfaces that allow the retrieval of GEO series with search capabilities that are superior to those offered natively by GEO while GEOMetaCuration allows the collaborative, manual curation of existing attributes (22). In addition, recent works also focused on mining GEO metadata, include ALE (23) and CEDAR (24). However, these works do not contemplate the automated identification of new attributes from each series’ existing metadata on GEO, particularly the number of time points and disease under investigation in each study.

In this work, we employed text mining techniques to develop Restructured GEO (ReGEO), a novel database to maximize re-use of GEO data by providing time series information about experimental data stored in GEO. We used a rule-based text mining algorithm to parse the metadata in GEO to automatically identify the number of time points in the experimental design. Our approach reached an accuracy rate of 93.5% and is entirely automatic. Our work demonstrates the utility of text mining in improving the usability of publicly available data. The ReGEO database can be accessed at http://www.regeo.org.

## Database description

### Data source and workflow

**Figure 2:**
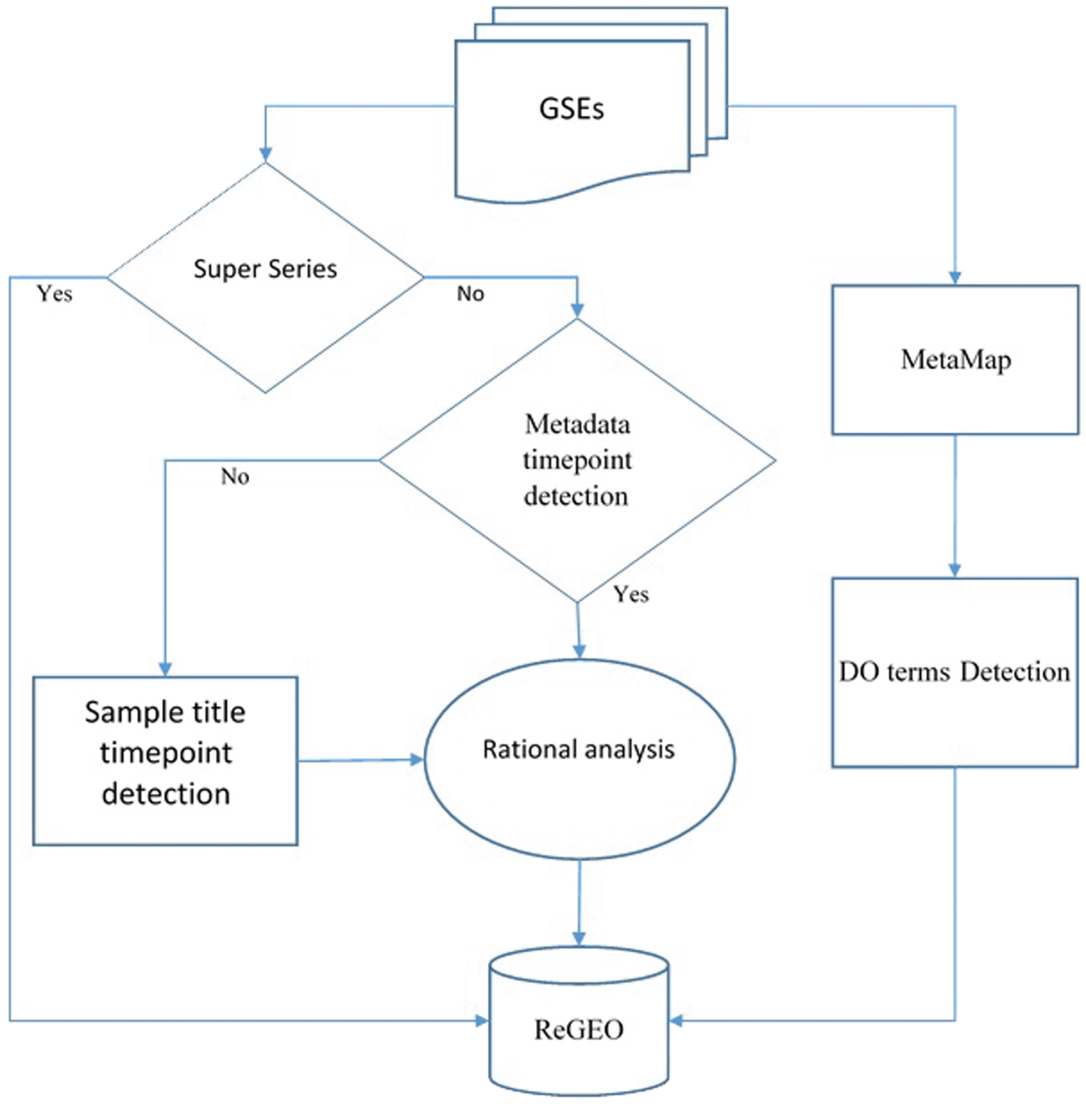
Workflow for developing ReGEO.

We obtained metadata and the titles of samples for Gene Series Expression (GSE) data from GEO and GEOmetadb (14). Among these data sets, super series datasets are a combination of a number of related datasets that will be analyzed individually. After excluding these super series from time point detection, the metadata and the titles of the samples were then analyzed by our novel natural language processing (NLP) search engine in order to detect the number of time points in each experiment. The resulting time points were subject to a rational analysis that validates the information of time points from both the metadata and the sample IDs (see next section for details). MetaMap was also applied to the metadata, and the Disease Ontology (DO) terms were detected from the annotated text for each dataset. Both the resulting time point information and DO were stored in the ReGEO database. The workflow is shown in Figure 2.

### Automated identification of time points

We designed an NLP text mining algorithm to detect information about the number of time points (for example `12 time points’, `7 developmental stages’) of gene expression data in GEO in the `Summary’ and `Overall design’ fields; listings of time points (for example `harvest at 6, 12 hours and 1, 3, 5 days’); and time-related statements, e.g. `early stage’ or `middle age’. In addition, the algorithm looks for the time point information in the titles of the series’ samples, taking into account that the time values may appear as a number followed by a time unit, or vice versa (e.g. `day 1’, `10 hr’). In summary, the algorithm evaluates the following four possible scenarios for determining the time points

1. Explicit statement of the number of time points.
2. Listing of time points.
3. Approximate statement.
4. Time points in titles of GSM samples.

A regular expression-based method was used to parse the GEO metadata to match the above patterns, followed by verification of consistence and rationality in the following conditions:

1. For time points expressed in the `Summary’ or `Overall design’ fields, the following four situations are considered as valid statement of time points.
  - Explicitly stated number of time points, time series or stages, and so on;
  - A list of numbers only in ascending order, followed by or starting with a time unit;
  - Multiple lists, as stated in b;
  - A list of numbers each with time unit.
2. Rational analysis for single-letter time-unit time points expressed in the titles of samples, the following rules are applied.
  - Treated as false time points if the same number is mentioned in summary or overall design with samples or patients.
  - Treated as false time points if certain other letters that are deemed irrelevant to time occurred in the same positions in other titles.
  - At least part of the time points in that dataset must show a regular pattern, either in an arithmetic progression or geometric progression series.

When filtering GEO series by a fixed number of time points, false negatives are less desirable than false positives because the latter can be easily identified and discarded during analysis. For this reason, our algorithm was designed to err by excess and, possibly, assign extra time points to a series rather than missing any of them.

The above text mining rules were fine-tuned on 600 manually-curated GEO series with ≥8 time points and evaluated on an additional 200 GEO series.

### Automated identification of diseases

The automated identification of diseases in each GEO series was accomplished with MetaMap and DO (25), where DO is a well maintained, standardized ontology for human disease with the purpose of providing the biomedical community with consistent, reusable and sustainable descriptions of human disease terms, phenotype characteristics and related medical vocabulary disease concepts (disease-ontology.org). MetaMap is a highly configurable program to map biomedical text to the UMLS Metathesaurus or, equivalently, to discover Metathesaurus concepts mentioned in text (26, 27). MetaMap uses a knowledge-intensive approach based on symbolic, NLP and computational-linguistic techniques. Besides being applied for both information retrieval and data-mining applications, MetaMap is one of the foundations of NLM’s Medical Text Indexer, used for semiautomatic and fully automatic indexing of biomedical literature at NLM.

We applied MetaMap to annotate the metadata of each GEO series, including `Title’, `Summary’, `Organism’ and `Overall design’ field, as well as the abstract of the citation, then we detected DO terms in the processed text, and labeled each GEO series with DOID_termIDs, DOID_termName, DOID_isLeaf, DOID_distancefromRoot and DOID_isObsolete. DOID_termIDs is the numeric identifiers of the DO term; DOID_termName is the corresponding DO terms; DOID_isLeaf indicates whether the matched term is a ‘leaf’ node in DO’s hierarchy t; DOID_distancefromRoot denotes the distance of matched term from the root in DO; DOID_isObsolete denotes if the term has become obsolete in the latest version of DO. The last three fields will ensure that the children of a matching node, if any, are also returned when a user searches for GEO series associated with a particular disease.

### The ReGEO database

ReGEO contains one record for each GEO series published on or before June 2018 and is updated on a monthly basis in order to incorporate the most recent submissions to GEO. ReGEO provides the number of time points and the time values extracted by our text mining algorithm from the metadata obtained from GEOmetadb. Descriptive information obtained from GEO such as the study’s title, type of experiment, and organism used is also included. Each record also contains the following structured information obtained from GEOmetadb (14) (version 3.5, for data up to March 2017): platform_id, platform_organism, platform_taxid, sample_organism, sample_taxid, contributor, contact, gse, last_update_date, pubmed_id, submission_date, title and type. Additional new data from March to June 2017 were directly obtained from GEO. We also used MetaMap program (26) to annotate the GEO series with DO (25, 28) and stored the information in ReGEO database.

**Figure 3:**
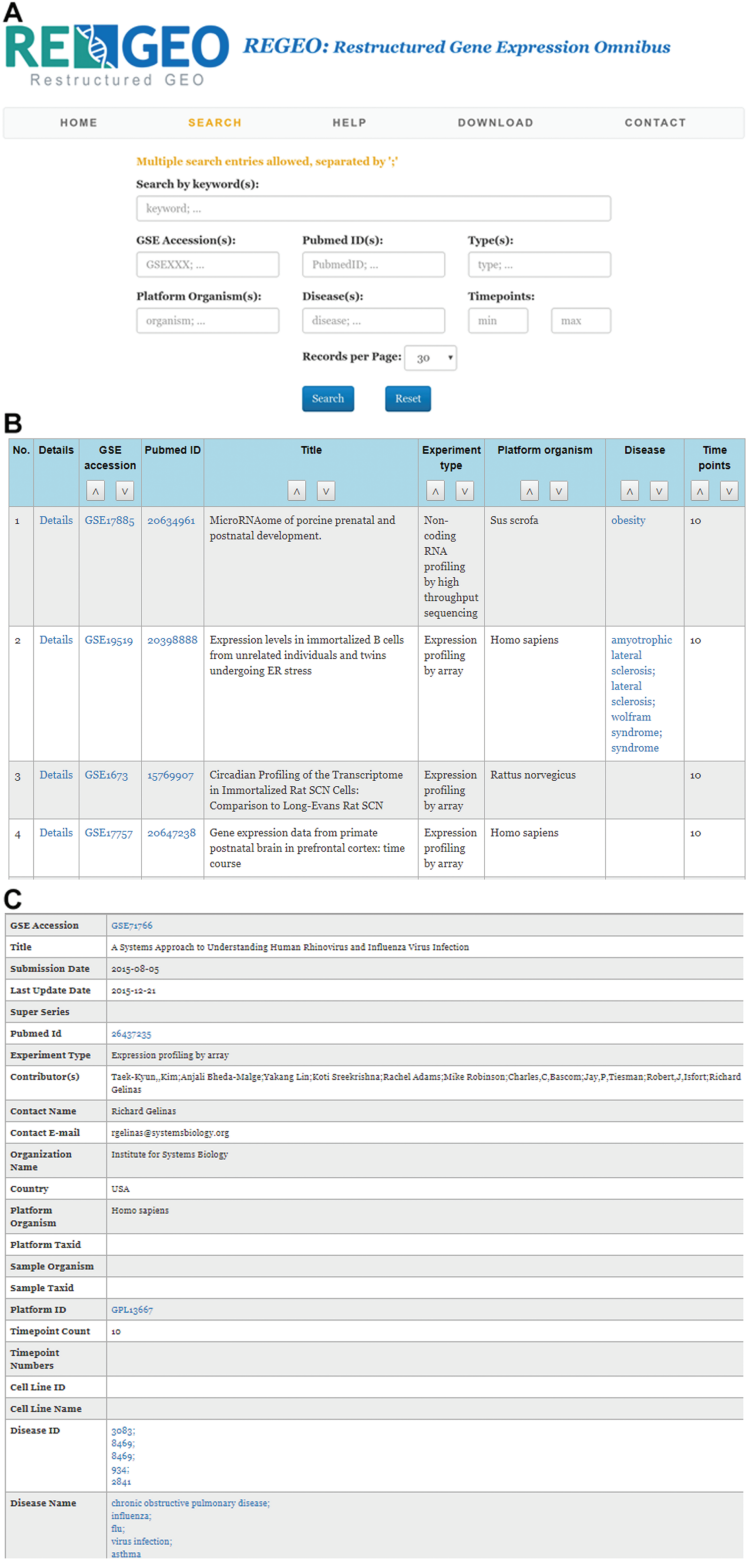
An illustration of the ReGEO database. a) The interface of ReGEO. b) Part of the search results from ReGEO database for GEO series with ten or more time points. c) An example of a data set stored in ReGEO that annotated with specific DO terms (“influenza” and “flu”) and their corresponding DOIDs (8469).

The web interface of ReGEO allows the search of GEO series by disease name, number of time points, and date of last update as illustrated in Figure 3a. The search result lists the accession numbers of the matching GEO series, their titles and the time points they contain, with accession numbers linked to the full information of the corresponding GEO series (Figure 3b). Clicking on an accession number (e.g. GSE55268) will bring up a page that displays the full information of the corresponding GEO series, as shown in Figure 3c.

### Evaluation and accuracy of metadata curation

We estimated the error rate of our text mining procedure by applying our algorithm to a test set of 200 randomly selected GEO series published on or before April 24, 2017. Each of these series consists of at least one GEO sample record with at least one time point. We manually identified the number of time point(s) of these 200 test series, out of which 159 series have one single time point, 33 have between 2 and 7 time points (inclusive), and 8 have over 7 time points.

Using this data set with manually-identified time point(s) as benchmark, our text mining program precisely identified the correct number of time point(s) in 167 out of the 200 series (83.5%). Due to inconsistent ways of reporting the baseline time point, it is common to have a small discrepancy of one time point between even two human curators; hence, we consider the computer-curated information on time points as correct if it is within ±1 time point as compared with the ground truth collected by human curators. Based on this criterion, our tests showed that our automated identification of time points has an accuracy rate of 93% (186/200). The error rate in series with a single time point is 1.26% (2/159), in series with 2–7 time points is 27.3% (9/33) and in series with over 7 time points is 37.5% (3/8). This evidences that the algorithm exhibits greater accuracy as the actual number of points of the series decreases, a satisfactory trait to correctly eliminate single time point data and to identify multiple time-points data for genome dynamic analysis.

The decreased accuracy for multiple time-points data is due to the increased complexity of these data sets—studies with two or more time points are generally less homogeneous and consistent in the way they refer to the time points in the experiment. One frequent case occurs when the series refer to an experiment that uses different time lengths in a treatment. For example, in series GSE28435, the correct number of time points is 6 (0, 0.25, 1, 3, 6, and 24 h). However, the treatment samples also included the seizure latency lengths of 4 min, 8 min, 9 min, 13.5 min, etc. For this reason the current version of the algorithm assigns 16 time points to this series. This kind of error is very hard to avoid and even human curators sometimes can make the same mistake in these cases.

The situation is getting even more complicated when the title of a sample includes time-related descriptors such as patient age or length of recovery time that do not refer to time points. This is illustrated by series GSE10288, where the ages of different patients were interpreted by the algorithm as time points. Similarly, the same samples under different stimulations measured on different time series also can cause the same problem. Grouping samples with patient ID or treatment could possibly help to avoid this problem. However, GEO metadata is not currently organized in this manner and future attempts to do this grouping could result in a tendency to label samples with a smaller number of time points, which would be undesirable.

Despite all these complications, the accuracy of our algorithm to identify single and low number of time points data ensure us to eliminate these data sets as they consist a majority of data in GEO database. The errors incurred by our method for high time-point data can then be quickly checked and fixed by a human due to the small number of these data sets. The current version of the algorithm to identify time ponts is optimized, balancing the trade-offs of precison and recall, and used to build ReGEO.

A similar approach was followed to evaluate the accuracy of the disease tags assigned to each series. From the randomly-selected 200 series that were manually curated, 172 were correctly labeled with the related diseases by the MetaMap method. The remaining 28 series were labeled with incorrect diseases or with no diseases at all. Therefore, the overall accuracy rate achieved was over 86%.

In order to be inclusive but precise, we have crafted Disease Search in a careful way. For example, to search a DO term, we consider title, summary, overall design and citations. We also used the advanced term mapping software, MetaMap, to allow partial match and ignored short abbreviation and stop words. In comparison, we do not impose any of these restrictions on Keyword search.

The full detail of these tests can be found in supplementary file ReGEO_test_results.csv (inserted as an attachment at the end of this document).

## Conclusions and perspectives

Identifying, pooling and harmonizing `small data’ from many studies is one of the goals of Big Data research, which will help investigators to conduct integrative analyses of a large number of data sets under similar experimental conditions without generating new data. The ultimate goal of ReGEO is to provide end users with a convenient and accurate way to identify and categorize data for their integrative data analysis.

Employing text mining techniques represents a new direction to achieve this goal by extracting useful information from unstructured metadata text. As such, ReGEO is designed not as a data `dump site’, but as a user-friendly database for data identification and integrative research. In this paper we have focused on identifying the number of time points and related diseases from the GEO unstructured data description texts as a starting point, making it possible to study gene expression dynamics across different data sets and under different experimental conditions.

Employing ontology, e.g. DO in our case for data annotation, could further facilitate data discovery and integrative analysis. For example, the number of detected and manually confirmed cases for `prostate cancer’ in ReGEO is 1444, in which only 1244 are consistent with the annotation in GEO database, and 82 are not present. However, several technical difficulties need to be further investigated. For one, there may exist many synonyms for ontology terms, and an efficient method is needed to organize and integrate these synonyms. Another difficulty is that ontology terms could lack sufficient detail. For example, the finest DO term on influenza infection is `Influenza’, which does not contain sub-terms on the specific trains of influenza virus. This issue can be ameliorated by integrating other ontologies, such as the Infectious DO, into the annotation. These limitations could be overcome by collaborating with ontology developers.

In the future, we aim to further extend the methods developed in this work by employing advanced text mining, NLP, machine learning, and ontology techniques (29), and applying these methods to identify and curate additional attributes and metadata from the unstructured texts provided by GEO. These methods, together with the related data analysis and visualization tools developed and integrated with ReGEO, can be applied to other public database for data discovery and integrative analysis.

## Supplementary Material

Supplementary Data

## Notes

**Database URL:** http://www.regeo.org
